# FF-STGCN: A usage pattern similarity based dual-network for bike-sharing demand prediction

**DOI:** 10.1371/journal.pone.0298684

**Published:** 2024-03-07

**Authors:** Di Yang, Ruixue Wu, Peng Wang, Yanfang Li

**Affiliations:** 1 School of Computer Science and Technology, Changchun University of Science and Technology, Changchun, China; 2 Jilin Provincial Joint Key Laboratory of Big Data Science and Engineering, Changchun, China; 3 Chongqing Institute of Changchun University of Science and Technology, Chongqing, China; Inner Mongolia University, CHINA

## Abstract

Accurate bike-sharing demand prediction is crucial for bike allocation rebalancing and station planning. In bike-sharing systems, the bike borrowing and returning behavior exhibit strong spatio-temporal characteristics. Meanwhile, the bike-sharing demand is affected by the arbitrariness of user behavior, which makes the distribution of bikes unbalanced. These bring great challenges to bike-sharing demand prediction. In this study, a usage pattern similarity-based dual-network for bike-sharing demand prediction, called FF-STGCN, is proposed. Inter-station flow features and similar usage pattern features are fully considered. The model includes three modules: multi-scale spatio-temporal feature fusion module, bike usage pattern similarity learning module, and bike-sharing demand prediction module. In particular, we design a multi-scale spatio-temporal feature fusion module to address limitations in multi-scale spatio-temporal accuracy. Then, a bike usage pattern similarity learning module is constructed to capture the underlying correlated features among stations. Finally, we employ a dual network structure to integrate inter-station flow features and similar usage pattern features in the bike-sharing demand prediction module to realize the final prediction. Experiments on the Citi Bike dataset have demonstrated the effectiveness of our proposed model. The ablation experiments further confirm the indispensability of each module in the proposed model.

## Introduction

The bike-sharing system represents an environmentally sustainable mode of transportation for short-distance urban travel, contributing to the reduction of carbon emissions and enhancing connectivity with public transit networks. Governments in cities such as New York, Washington, Beijing, and Shanghai are actively advocating for bike-sharing programs to mitigate traffic congestion. It is reported, during the first half of 2021, New York City witnessed a daily average bike-sharing usage of 5 million times. An effective allocation strategy enhances user experience and generates revenue. Conversely, poor, or imbalanced allocation strategies markedly diminish operational efficiency, raise dispatch costs, and lower user satisfaction. Bike-sharing demand prediction is a prerequisite for effective allocation strategies. It is analyzing the law of borrowing and returning bikes at stations to predict the bike-sharing demand soon. By capturing the dynamics of bike-sharing demand, operators can optimize allocation strategies, thereby creating an efficient, cost-effective, and user-friendly bike-sharing system.

Bike-sharing demand prediction models face several primary challenges: Initially, the complex spatio-temporal dependency is a major factor influencing the accuracy of bike-sharing demand prediction and concurrently reflects user travel patterns [1]. Investigating the local spatio-temporal characteristics of a specific station within a bike-sharing system fails to comprehensively capture the overall user travel patterns of the entire system, resulting in a decline in predictive model performance. In the spatial dimension, bike-sharing demand between adjacent stations mutually influences each other, displaying similar user travel patterns. Similarly, stations within the same functional zone may also exhibit comparable user travel patterns [2]. In the temporal dimension, bike-sharing demand exhibits continuous features. Therefore, the analysis of user travel patterns and the learning of spatio-temporal features pose significant challenges for bike-sharing demand prediction.

Furthermore, bike usage trends in bike-sharing demand are influenced by various factors [3]. These trends exhibit both randomness and dynamism, making prediction a highly complex task. Factors affecting bike-sharing demand prediction can be categorized into internal and external factors. Internally, short-term (continuity) and long-term (daily and weekly periodicity) aspects impact bike-sharing demand prediction. Users’ borrowing and returning behaviors between bike-sharing stations contribute to the time delay in bike-sharing demand. Externally, the POI and morning and evening peak hours significantly impact variations in bike-sharing demand. For example, stations near residential areas experience increased demand during morning peak hours when public travel rises. Therefore, the analysis of the intrinsic spatio-temporal features of bike-sharing enables the uncovering of global spatio-temporal patterns in demand. Additionally, introducing external influencing factors helps excavate hidden correlations between stations. This understanding facilitates an accurate prediction of bike-sharing demand by comprehending user travel patterns.

To address these challenges, this study proposes a spatio-temporal bike-sharing demand prediction model (FF-STGCN) based on usage pattern similarity analysis. The model captures correlated features among stations, mitigating the limitations in multi-scale spatio-temporal accuracy. The model adopts the idea of feature integration, constructing a multi-scale spatio-temporal feature fusion module based on a multi-scale feature attention (MS-FA) network and an attention-based feature fusion network. This approach minimizes the loss of multi-scale spatio-temporal features. Subsequently, a bike usage pattern similarity learning module is developed, utilizing temporal and spatial similarity calculators to capture underlying correlated features among stations. In conclusion, the proposed bike-sharing demand prediction model employs a dual network structure containing a flow-based feature learner (FFL) and a pattern-based feature learner (PFL), aggregated to enhance bike-sharing demand accuracy.

## Literature review

Accurately predicting the bike-sharing demand is a crucial foundation for ensuring the effective operation and management of bike-sharing systems, and it has garnered widespread attention in recent years. Bike-sharing demand prediction can be categorized into two methods based on the prediction task: cluster-based and station-based.

The cluster-based prediction methods utilize clustering algorithms such as hierarchical clustering [4, 5], Gaussian mixture model, supervised clustering [6], and community detection algorithms [7–10], etc. By analyzing different indicators, these methods reveal the correlations between stations to achieve the prediction of bike-sharing demand. For example, to capture the connections between bike-sharing stations, Wang et al. designed a two-tier fuzzy C-means clustering algorithm. This algorithm clusters bike-sharing stations into groups by combining the geographic location information of the stations and the migration trends of bikes between them. Subsequently, they integrated a multi-similarity reference model to predict the demand for bike-sharing within each group [11]. Gu et al. have proposed an interpretable bike flow prediction (IBFP) method. This approach involves dividing the city into regions based on flow density and utilizing subspace clustering to group these regions, constructing interpretable patterns for bike-sharing flow. Subsequently, the method models spatio-temporal interactions using graph regularized sparse representation to predict bike-sharing flow patterns [12]. However, these methods lack solutions to address the complex iterative problems, leading to instability in trend iterations during clustering. Therefore, some researchers have proposed iterative optimization solutions to tackle this issue. For instance, Zhao et al. introduced a hyper-clustering algorithm designed to capture mobility trends among individuals and clusters, enhancing the spatio-temporal neural network for demand prediction in bike-sharing systems [13]. Existing research has thoroughly demonstrated the effectiveness and accuracy of cluster-based prediction methods. However, these methods rely on random initialization or manual parameter setting, leading to potential uncertainty in the resulting clustering outcomes. Furthermore, cluster-based approaches inadequately consider variations in demand among individual sites within the clusters, potentially limiting their ability to accurately predict bike-sharing demand.

In station-based prediction methods, the studies effectively predict bike-sharing demand at each station through the construction of a network model. Researchers employ machine learning to analyze historical data, discern patterns within it, and project future demand. Harikrishnakumar et al., for instance, introduced a method utilizing the Quantum Bayesian Network (QBN) framework for real-time analysis of bike-sharing demand, aiming to enhance both computational efficiency and accuracy [14]. However, it’s worth noting that bike-sharing demand predictions relying on machine learning typically necessitate a substantial amount of data, and the presence of incomplete or inaccurate data may result in a decline in accuracy. To address this, time series analysis models have been implemented in the prediction of bike-sharing demand. For instance, Leem et al. proposed a two-stage time series prediction model based on online learning to tackle the challenge of low prediction accuracy in environments with limited data and computational resources [15]. This model attains higher accuracy with fewer computational infrastructures. Additionally, the ARIMA model and its variants employ autoregressive or moving average models to capture the temporal autocorrelation of data [16–18]. Meanwhile, Cortez-Ordoñez et al. evaluated the significant distinctions among bike-sharing systems with diverse scales, characteristics, or usage patterns. They also conducted a detailed analysis of the performance of existing predictive algorithms, including ARIMA, Linear Models, and others, in each scenario [19]. Developments in Deep Learning have led to the widespread use of various deep learning models to extract spatio-temporal correlations for predicting bike-sharing demand, such as Convolutional Neural Network (CNN) [20], Long Short-Term Memory (LSTM) [21], and Recurrent Neural Network (RNN) with its variants [22, 23]. For instance, Li et al. used feature engineering techniques to enhance the data, and then employed LSTM to capture the spatio-temporal dependence of the historical data and make predictions [24]. And Chen et al. proposed a model for predicting bike-sharing demand that integrates Discrete Wavelet Transform (DWT), Autoregressive Integrated Moving Average (ARIMA), and Long Short-Term Memory neural network (LSTM). In detail, they decomposed the demand sequence into three high-frequency components and one low-frequency component using DWT. Subsequently, ARIMA and LSTM were applied for individual predictions. Lastly, the predicted results underwent reconstruction through DWT to establish the final prediction structure [25]. Furthermore, many scholars believe that combining different models can improve the accuracy of demand prediction for bike-sharing. Specifically, Bai et al. use a cascade graph convolutional recurrent neural network to extract spatio-temporal correlations between data and two multi-layer LSTM networks to represent external meteorological data and time meta separately [26]. Chai et al. produce a multi-view spatio-temporal framework to combine characteristics into one prediction model framework of predicting the bike-sharing demand [27]. Alternatively, some scholars have integrated GCN and attention mechanisms in a natural way to tackle the issue of incorporating irrelevant stations’ features in the prediction process because of inadequate or erroneous prior knowledge [28–31]. For example, Huang et al. developed the Temporal Multi-graph Convolutional (TMGCN) network to capture the spatial topologies contained in the dynamic OD graphs in terms of time and exploit the GAN structure to overcome the high sparsity of OD demands [32]. Furthermore, considering both the data collected from the bikes themselves and the extended analysis data provides valuable insights for constructing a network to predict the demand for bike-sharing [33, 34]. Unfortunately, user travel behavior varies across time and space, resulting in cyclical and volatile changes in bike-sharing supply and demand [35, 36]. The stochastic fluctuations of individual stations can interfere with the feature extraction and pattern learning of overall demand. These models are insensitive to random fluctuations and struggle to handle complex bike-sharing datasets, which leads to low prediction accuracy. Therefore, it is essential to mitigate the stochastic volatility of bike-sharing demand to improve the prediction accuracy and robustness of the models.

However, most studies typically utilize independent modules, such as convolutional networks, recurrent neural networks, and their variants, to separately capture temporal and spatial dependencies. These studies capture dependencies between temporal and spatial factors in an ordered manner, but they don’t fully consider the dynamic spatio-temporal dependencies of the system. As a result, they are unable to tackle the delay in bike-sharing demand caused by users’ dynamic borrowing and returning behavior. Moreover, in practical systems, infrequent user travel between adjacent stations results in a low correlation between them. Conversely, distant stations may display analogous user usage patterns, indicating an implicit correlation. Consequently, extant studies predominantly emphasize localized effects, neglecting the overarching system dependency and the stochastic nature of user borrowing and returning behavior. This disregard contributes to a diminished accuracy in prediction.

To comprehensively consider the randomness, global dependency of the bike-sharing system, and user behavior patterns, we propose a bike-sharing demand prediction model based on the similarity of user usage patterns.

## Problem definition

In this part, we define the mathematical symbols and provide detailed explanations of the problem at hand.

**Definition 1** (Inflow matrices) At the *t*^*th*^ time slot, we define the bike-sharing inflow matrices as *I*^*t*^. $I_{1,1}^{t}$ is the quantity of borrowing from station *s*_*j*_ and returning to station *s*_*i*_ during the *t*^*th*^ time slot.

$$\begin{array}{c} I^{t}=(\begin{array}{ccccc} I_{1,1}^{t} & I_{1,2}^{t} & \cdots & I_{1,n-1}^{t} & I_{1,n}^{t}\\ I_{2,1}^{t} & I_{2,2}^{t} & \cdots & I_{2,n-1}^{t} & I_{2,n}^{t}\\ \vdots & \vdots & \ddots & \vdots & \vdots \\ I_{n,1}^{t} & I_{n,2}^{t} & \cdots & I_{n,n-1}^{t} & I_{n,n}^{t} \end{array}) \end{array}$$ (1)

$$\begin{array}{c} I_{i,j}^{t}=\left|\left\{P\in {\text{P}}_{t}|(P(O),P(D))\in (s_{j},s_{i})\wedge (P(O),P(D))\notin (s_{j},s_{j})\right\}\right| \end{array}$$ (2)

where P_*t*_ denotes the trip in *t*^*th*^ time slot *P*(*O*) and *P*(*D*) represent the borrowing and return stations of a trip *P*. (*P*(*O*), *P*(*D*)) ∈ (*s*_*j*_, *s*_*i*_) ∧ (*P*(*O*), *P*(*D*)) ∉ (*s*_*j*_, *s*_*j*_) is a trip that borrows from the station *s*_*j*_, and comes back to another station except the station *s*_*j*_. |•| denotes the cardinality of a set.

**Definition 2** (Outflow matrices) At the *t*^*th*^ time slot, we define the bike-sharing inflow matrices as $O^{t}.O_{1,1}^{t}$ is the quantity of borrowing from station *s*_*i*_ and returning to station *s*_*j*_ during the *t*^*th*^ time slot.

$$\begin{array}{c} O^{t}=(\begin{array}{ccccc} O_{1,1}^{t} & O_{1,2}^{t} & \cdots & O_{1,n-1}^{t} & O_{1,n}^{t}\\ O_{2,1}^{t} & O_{2,2}^{t} & \cdots & O_{2,n-1}^{t} & O_{2,n}^{t}\\ \vdots & \vdots & \ddots & \vdots & \vdots \\ O_{n,1}^{t} & O_{n,2}^{t} & \cdots & O_{n,n-1}^{t} & O_{n,n}^{t} \end{array}) \end{array}$$ (3)

$$\begin{array}{c} O_{i,j}^{t}=\left|\left\{P\in {\text{P}}_{t}|(P(O),P(D))\in (s_{i},s_{j})\wedge (P(O),P(D))\notin (s_{i},s_{i})\right\}\right| \end{array}$$ (4)

where P_*t*_ denotes the trip in *t*^*th*^ time slot *P*(*O*) and *P*(*D*) represent the borrowing and return stations of a trip *P*. (*P*(*O*), *P*(*D*)) ∈ (*s*_*j*_, *s*_*i*_) ∧ (*P*(*O*), *P*(*D*)) ∉ (*s*_*i*_, *s*_*i*_) is a trip borrowing from the station *s*_*i*_, and returning in another station except the station *s*_*i*_. |•| is the cardinality of a set.

**Definition 3** (Station geographic characteristics) We construct the geographical features of the station *s*_*i*_ by utilizing the number of POI types in the region to which the station belongs, denoted as *P*_*i*_ = {*p*_1_, *p*_2_, …*p*_*M*_}. Here *p*_*m*_ denotes the value of the class *m* interest POI vector.

**Definition 4** (Station temporal sequences) We define the station spatial feature, based on the historical order data of bike-sharing, is $X_{i}^{T}=\{x_{i}^{1},x_{i}^{2},\ldots ,x_{i}^{t}\}$.

**Problem** (Prediction problem) We utilize the historical inflow and outflow features *I*^*T*−1^ = {*I*^0^, …, *I*^*t*−1^} and *O*^*T*−1^ = {*O*^0^, …, *O*^*t*−1^} until time slot *t*−1, as well as the station geographic characteristics of stations *S*, carry out supply and demand predictions for bike-sharing, i.e. ${\hat{y}}_{in}^{i}=\sum_{j=0}^{N}I_{i,j}^{t}$ and ${\hat{y}}_{out}^{i}=\sum_{j=0}^{N}O_{i,j}^{t}$, for any single station s during time period *t*. Can be shown Eq 5.

$$\begin{array}{c} \left({\hat{y}}_{in},{\hat{y}}_{out})=F(I^{T-1},O^{T-1},P\right) \end{array}$$ (5)

where *F*(•) denotes the prediction function of my model.

## Methodology

**Fig 1:**
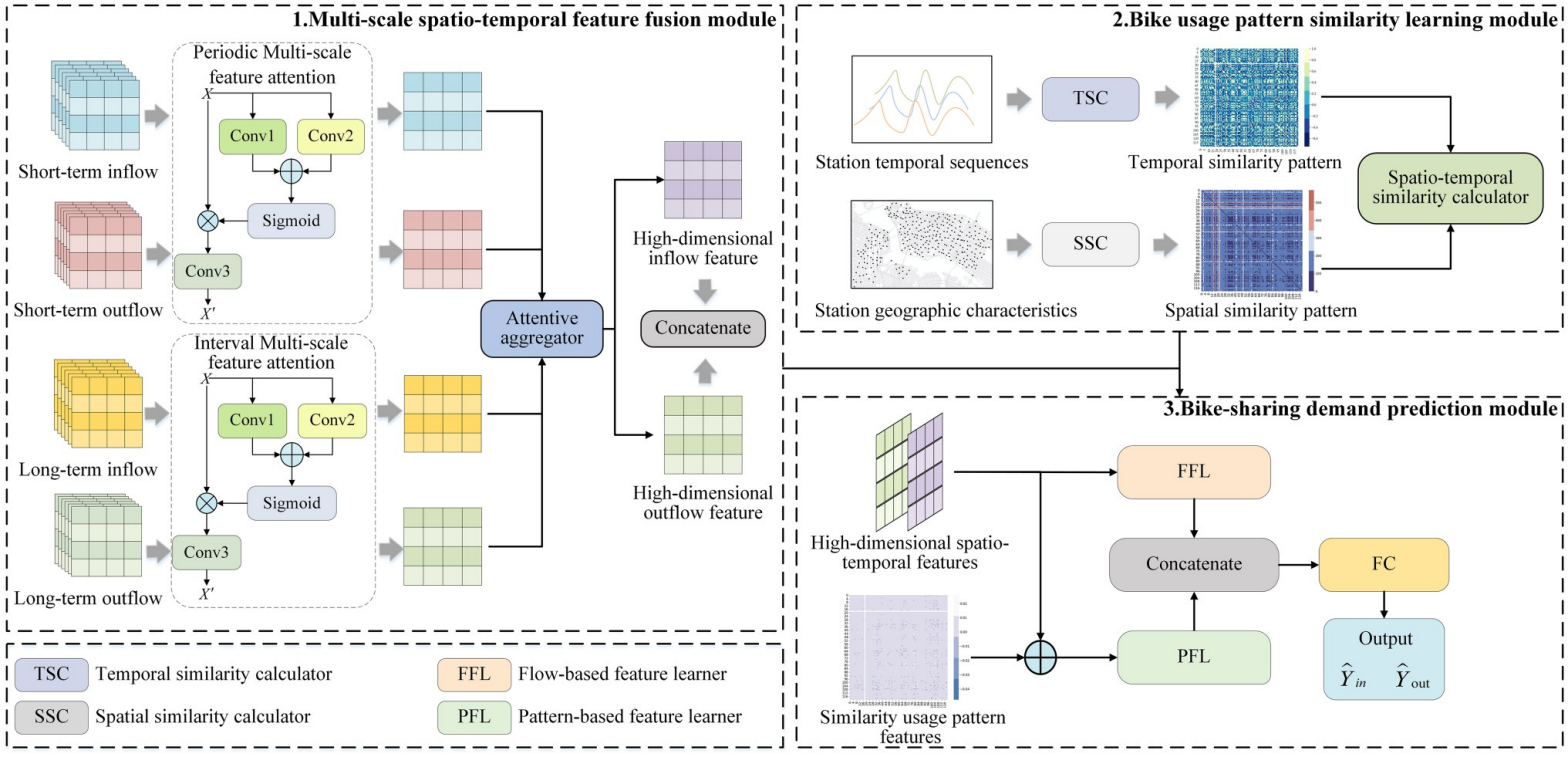
General structure of the proposed model.

Fig 1 illustrates the general structure of the proposed model, which consists of three modules: the multi-scale spatio-temporal feature fusion module, the bike usage pattern similarity learning module, and the bike-sharing demand prediction module. Specifically, the multi-scale spatio-temporal feature fusion module based on the idea of feature integration utilizes MS-FA network and the attention mechanism to address limitations in multi-scale spatio-temporal accuracy. Then, the concept of estimating similar demand is incorporated into the design of a bike usage pattern similarity learning module to obtain usage pattern information by capturing the underlying correlated features among stations. Finally, we develop a bike-sharing demand prediction module which use a dual network structure containing FFL and PFL to learn high-dimensional spatio-temporal features and similarity usage pattern features for realizing the final prediction.

### Multi-scale spatio-temporal feature fusion

The demand for bike-sharing exhibits strong spatio-temporal characteristics. Intuitively, the demand is influenced by short-term dependencies on recent historical flow data, while also exhibiting daily periodicity dependencies (long-term dependencies). However, when modeling objects at different scales using existing methods, a series of pooling layers or other cross-layer operations can result in the loss of features. To mitigate this loss of multi-scale spatio-temporal features, we have designed a multi-scale spatio-temporal feature fusion module. This module helps to identify and utilize the periodicity of bike-sharing demand, thereby improving the accuracy of the prediction model.

The multi-scale spatio-temporal feature fusion module consists of two parts: feature training and feature fusion. To identify and utilize the periodicity of bike-sharing demand, feature training develops a dual MS-FA network to train short-term and long-term features. Then, an attention-based feature fusion is designed to obtain high-dimensional spatio-temporal features by fusing multi-scale features.

**Fig 2:**
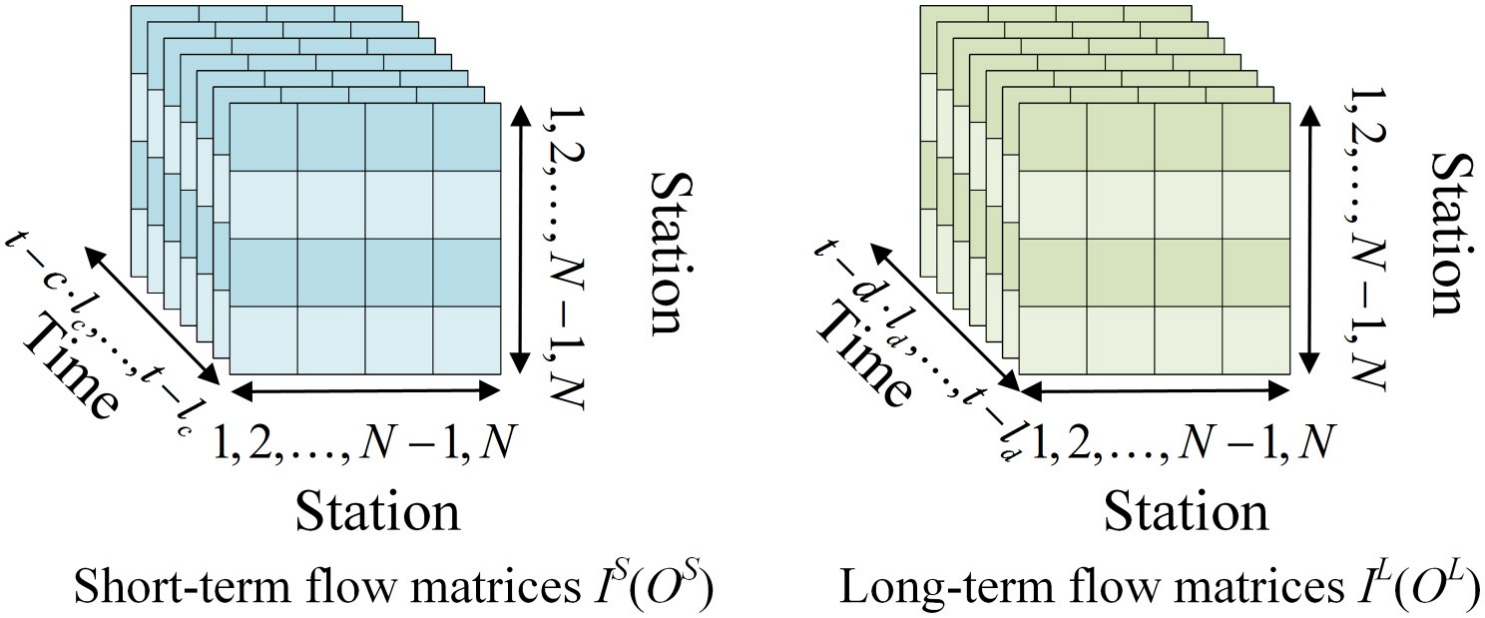
Short-term and long-term flow matrices.

Primarily, to consider both short-term and long-term dependencies systematically, we take temporal features pertaining to short-term and long-term considerations into the model inputs. The inflow and outflow matrices (*I*^*t*^ and *O*^*t*^) expanded into distinct entities, namely, short-term inflow and outflow matrices (*I*^*S*^ and *O*^*S*^), and long-term inflow and outflow matrices (*I*^*L*^ and *O*^*L*^), as depicted in Fig 2.

In this context, *t* represents the predicted time point, *N* denotes the number of predicted stations, *c* represents the number of consecutive time series for short-term dependencies, and *d* denotes the number of consecutive days for long-term dependencies. *l*_*c*_ signifies the continuous temporal interval, while *l*_*d*_ characterizes the daily temporal interval. The dependency for *l*_*c*_ is delineated as follows:

$$\begin{array}{c} l_{d}=T_{day}/l_{c} \end{array}$$ (6)

where *T*_*day*_ denotes 24 hours of a whole day.

#### Feature training

**Fig 3:**
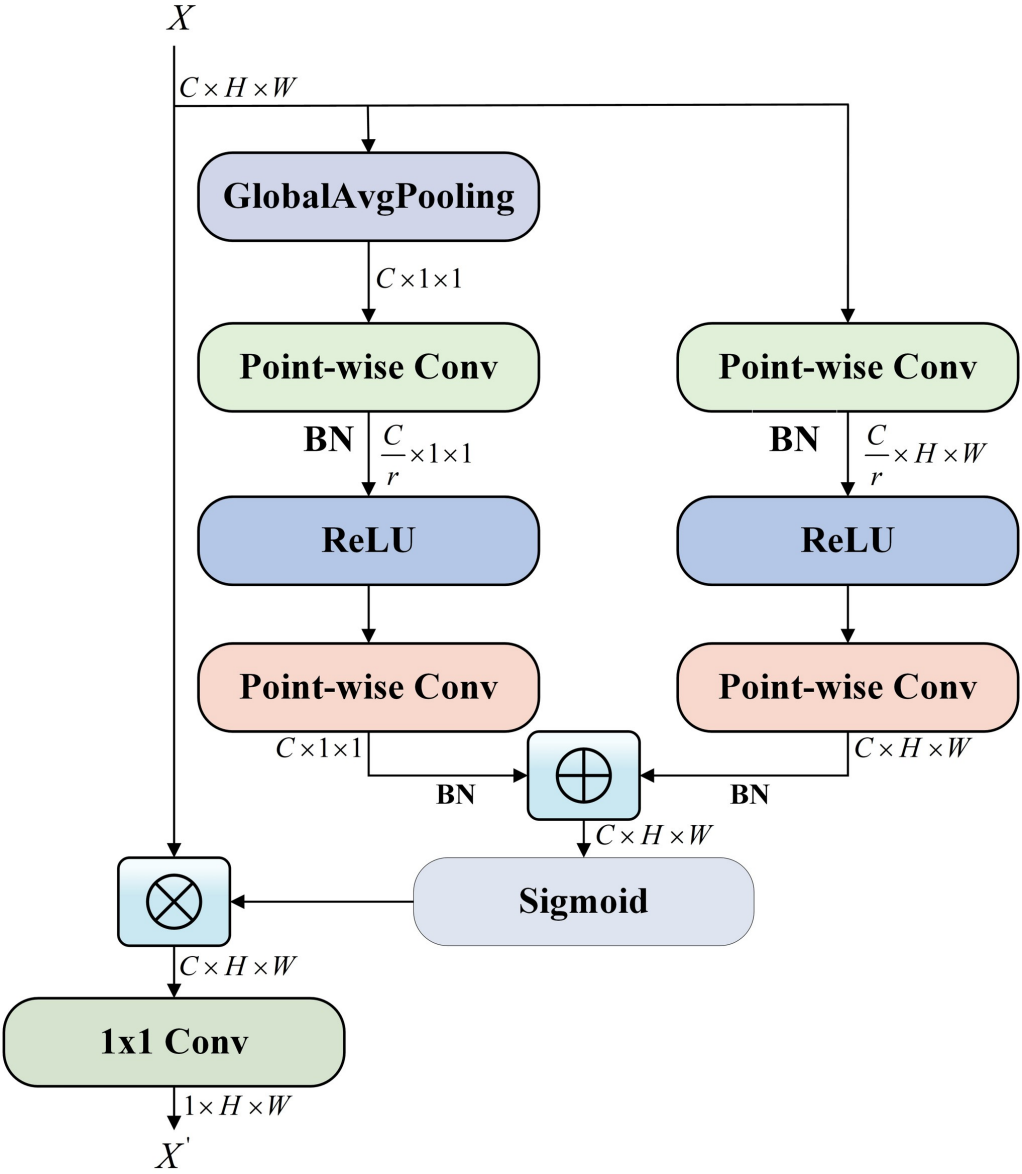
Multi-scale feature attention (MS-FA) network.

Capturing the characteristics of bike-sharing demand across various time slots is crucial for enriching the bike-sharing demand features. Therefore, we have constructed a dual MS-FA network in the feature training process. This network includes both periodic MS-FA and interval MS-FA to train long-term and short-term flow matrices separately. The main structure of the MS-FA network is shown in Fig 3.

The underlying principle of MS-FA is that feature attention can be achieved at various scales by adjusting the size of the spatial pooling operation. Specifically, Global average pooling (GAP) and local channel context aggregator are used to capture the global and local contexts of the short-term inflow matrix separately. Then, the local context is added to the global context in the attention module for feature fusion purposes, enriching temporal information. Finally, 1 × 1 convolution kernels are applied on flow matrices to integrate the features at short-term and long-term. Among them, point-wise convolution (PWConv) is chosen as the local channel context aggregator, which utilizes point-wise channel interactions at each spatial location.

Therefore, we apply MS-FA on short-term inflow matrices to obtain the short-term inflow embedding:

$$\begin{array}{c} {\hat{I}}^{S}=\sigma (\sum_{i=1}^{h}w_{i}*I_{\text{i}}^{S^{\prime }}+b_{i}) \end{array}$$ (7)

The learnable parameters are denoted as *w*_i_ and *b*_*i*_. And the convolution operator is denoted as *. Then the ReLU activation function is *σ*(•). $I^{S^{\prime }}$ is computed by:

$$\begin{array}{c} I^{S^{\prime }}=I^{S}⊗M(I^{S})=I^{S}⊗\sigma (L(I^{S})⊕g(I^{S})) \end{array}$$ (8)

where $M(I^{S})\in R^{h\times N\times N}$ is the attentional weight generated by MS-FA. ⊕ denotes the broadcasting addition. And ⊗ denotes the element-wise multiplication. The local channel context $L(I^{S})\in R^{h\times N\times N}$ and global channel context $g(I^{S})\in R^{h\times N\times N}$ are computed by:

$$\begin{array}{c} \left(I^{S})=\text{B}(PWConv_{2}(\delta (\text{B}(PWConv_{1}(I^{S}))))\right) \end{array}$$ (9)

$$\begin{array}{c} g(I^{S})=GAP(\text{B}(PWConv_{2}(\delta (\text{B}(PWConv_{1}(I^{S})))))) \end{array}$$ (10)

where the kernel sizes of *PWConv*_1_ is $\frac{h}{\text{r}}\times h\times 1\times 1$, it reduces the original input feature’s channel count by $\frac{1}{r}$. B(•) denotes Batch Normalization is used to accelerate feature convergence. *δ*(•) is the ReLU activation function. The kernel sizes of *PWConv*_2_ is $h\times \frac{h}{r}\times 1\times 1$, which is utilized for the purpose of reinstating the original number of channels’ features.

Similarly, the long-term inflow ${\hat{I}}^{L}\in R^{N\times N}$, short-term outflow ${\hat{O}}^{S}\in R^{N\times N}$ and long-term outflow ${\hat{O}}^{L}\in R^{N\times N}$ embeddings are computed similar from Eqs 7 to 10.

#### Feature fusion

Feature training is helpful to further learning characteristics of bike-sharing demand across various time slots. Meanwhile, the demand also exhibits limitations in multi-scale spatio-temporal accuracy due to a series of convolution operations that produce coarse-grained results. Hence, to overcome the potential constraints of bike-sharing demand and improve prediction performance, we have developed a feature fusion method that fuses both short-term and long-term features.

In feature fusion, we propose a fusion network that leverages the attention mechanism to combine short-term and long-term features, thereby extracting high-dimensional inflow features defined as follows:

$$\begin{array}{c} \hat{I}=\beta_{\text{I}}^{S}\cdot {\hat{I}}^{S}+\beta_{\text{I}}^{L}\cdot {\hat{I}}^{L} \end{array}$$ (11)

$\beta_{\text{I}}^{S}$ and $\beta_{\text{I}}^{L}$ are computed by:

$$\begin{array}{c} \beta_{I}^{S}=\frac{\text{exp}(\sum_{i=1}^{N}\omega_{i}\cdot {\hat{I}}_{i}^{S})}{\text{exp}(\sum_{i=1}^{N}\omega_{i}\cdot {\hat{I}}_{i}^{S})+\text{exp}(\sum_{i=1}^{N}\omega_{i}\cdot {\hat{I}}_{i}^{L})} \end{array}$$ (12)

$$\begin{array}{c} \beta_{I}^{L}=\frac{\text{exp}(\sum_{i=1}^{N}\omega_{i}\cdot {\hat{I}}_{i}^{L})}{\text{exp}(\sum_{i=1}^{N}\omega_{i}\cdot {\hat{I}}_{i}^{S})+\text{exp}(\sum_{i=1}^{N}\omega_{i}\cdot {\hat{I}}_{i}^{L})} \end{array}$$ (13)

where the learnable parameter is *ω*_*i*_. Similarly, we have the high-dimensional outflow features defined as follows:

$$\begin{array}{c} \hat{O}=\beta_{\text{O}}^{S}\cdot {\hat{O}}^{S}+\beta_{\text{O}}^{L}\cdot {\hat{O}}^{L} \end{array}$$ (14)

where $\beta_{\text{O}}^{S}$ and $\beta_{\text{O}}^{L}$ are computed similarly to Eqs 12 and 13.

Finally, to jointly consider the demand and supply features, we connect the outputs mentioned above:

$$\begin{array}{c} \text{T}=(\hat{I}∥\hat{O}) \end{array}$$ (15)

where ∥ denotes the concatenation operation. The high-dimensional spatio-temporal feature is used as $\text{T}\in R^{2\times N\times N}$.

### Bike usage pattern similarity learning

In bike-sharing system, demand is influenced by a variety of dynamic factors, including the geographical environment and the unpredictable borrowing and returning behavior of users. These factors contribute to the random volatility of bike-sharing characteristics. However, stations with similar bike usage patterns can reflect the borrowing and returning records of other stations in the same category during the same period. Hence, identifying stations with similar bike usage patterns to reveal potential connections in bike-sharing and mitigate the impact of stochastic volatility on feature learning is crucial for improving predictive performance.

The bike usage pattern similarity learning module, which is applied to obtain similarity usage pattern features, is composed of three parts: temporal similarity calculator, spatial similarity calculator, and spatio-temporal similarity calculator. The temporal similarity calculator is leveraged, which uses a metric, namely Dynamic Time Warping (DTW) [37], to calculate the similarity of bike usage patterns between stations in the temporal dimension. Then, to calculate the similarity of bike usage patterns in the spatial dimension, we develop a spatial similarity calculator using the Pearson Correlation Coefficient [38]. Finally, the spatio-temporal similarity calculator is introduced to fuse the similarity of spatial bike usage patterns and similarity of temporal bike usage patterns to construct the similarity usage pattern feature.

#### Temporal similarity calculator

The temporal features of bike-sharing demand are crucial dynamic factors in analyzing the usage patterns of bikes at stations. Over the same time period, the usage patterns of some bike-sharing stations may exhibit similarities. Identifying stations that have similar bike usage patterns over a period of time and using these stations to reflect the borrowing and returning records of other stations in the same category during the same period would be beneficial for developing bike-sharing demand prediction. The DTW algorithm overcoming the constraint of requiring time series to have the same length when applying Euclidean distance, it has been widely used in subsequent research to measure the similarity between time series. Therefore, we utilize the DTW algorithm to calculate the similarity values of time series between any two stations within the preceding k time steps of the prediction moment, measuring their similarity in temporal patterns.

For example, We assume that the time series data *X*_*T*−*t*_ and *Y*_*T*−*t*_ for two stations, at *k* time steps before the predicted moment, are denoted as follows:

$$\begin{array}{c} \begin{array}{c} \left\{ \begin{array}{c} X_{T-t}=\{x_{t-1},x_{t-2},\ldots x_{t-k}\}\\ Y_{T-t}=\{y_{t-1},y_{t-2},\ldots y_{t-k}\} \end{array}\right. \end{array} \end{array}$$ (16)

**Fig 4:**
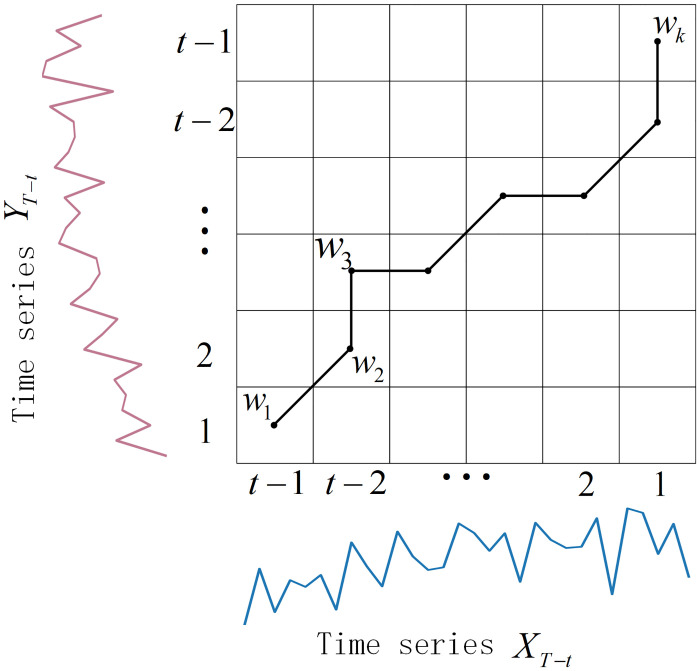
Dynamic time warping (DTW).

In the Dynamic Time Warping (DTW) algorithm, the initial step involves computing the distances between individual elements of the two time series to generate the cost matrix *D*, respectively:

$$\begin{array}{c} \begin{array}{c} D(X,Y)=(\begin{array}{ccc} d(x_{t-k},y_{t-k}) & \ldots & d(x_{t-k},y_{t-1})\\ \vdots & \ddots & \vdots \\ d(x_{t-1},y_{t-k}) & \cdots & d(x_{t-1},y_{t-1}) \end{array}) \end{array} \end{array}$$ (17)

$$\begin{array}{c} \begin{array}{c} d(x_{i},y_{j})=dist(x_{i},y_{j})+\text{min}(d(x_{i-1},y_{j-1}),d(x_{i},y_{j-1}),d(x_{i-1},y_{j})) \end{array} \end{array}$$ (18)

$$\begin{array}{c} \begin{array}{c} dist(x_{i},y_{j})=|x_{i}-y_{j}|,i=t-k,\ldots ,t-1,j=t-k,\ldots ,t-1 \end{array} \end{array}$$ (19)

where *dist*(*x*_*i*_, *y*_*j*_) represents the Euclidean distance between the nodes *x*_*i*_ and *y*_*j*_. Next, in the cost matrix *D*, find a path from the top-right to the bottom-left corner, where the sum of the values of the elements traversed is minimized. This is the warping path of time series *X* and *Y*, denoted as *W*(*X*, *Y*) = {*w*_1_, *w*_2_, …, *w*_*m*_}, *t* − 1 ≤ *m* ≤ 2*t* − 2. Fig 4 illustrates the execution process of the Dynamic Time Warping (DTW) algorithm described above. The squares in the figure represent the distance cost between two elements of the example time series, while the lines in the path depict the warping path connecting the two example time series.

Finally, by calculating the cumulative cost values, the similarity in usage patterns between two stations in the temporal dimension is measured to construct a time similarity matrix *TSV*, as shown in the following formula:

$$\begin{array}{c} \begin{array}{c} TSV=(\begin{array}{ccc} \infty & \ldots & tsv(s_{1},s_{n})\\ \vdots & \ddots & \vdots \\ tsv(s_{n},s_{1}) & \cdots & \infty \end{array}) \end{array} \end{array}$$ (20)

$$\begin{array}{c} \begin{array}{c} tsv(s_{i},s_{j})=\frac{\sqrt{\sum_{m=1}^{M}w_{m}}}{M} \end{array} \end{array}$$ (21)

**Fig 5:**
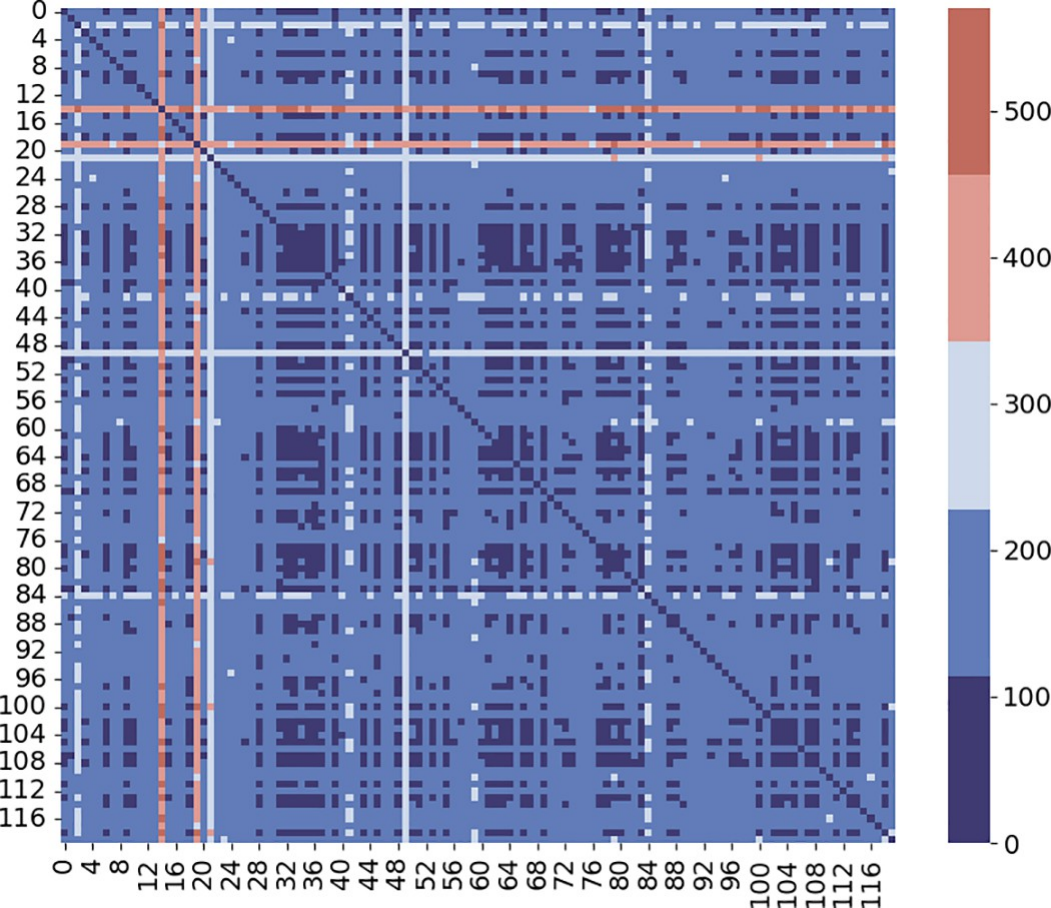
Temporal similarity between stations.

Fig 5 displays a heatmap illustrating the temporal similarity between stations over the time period from *t* − *k* to *t* − 1.

#### Spatial similarity calculator

**Fig 6:**
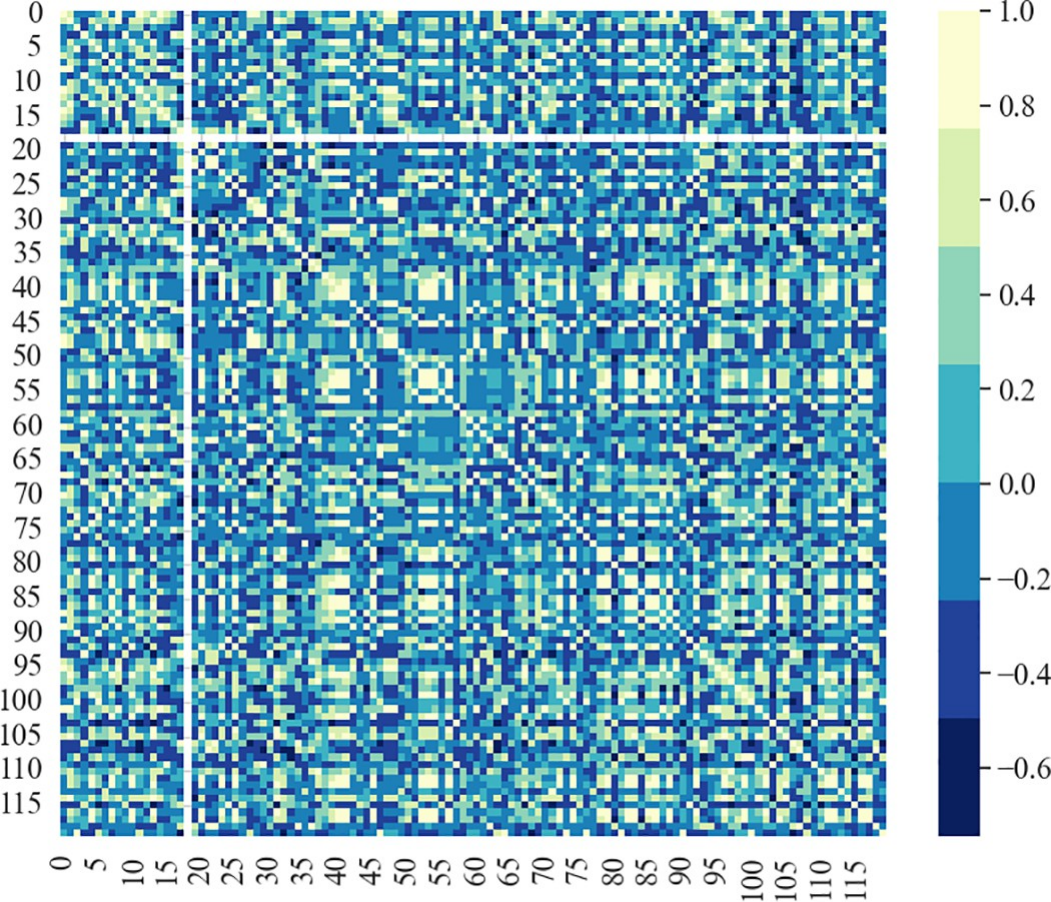
Spatial similarity between stations.

The geographical environment is a crucial spatial factor that is strongly correlated with user travel behavior and significantly influences bike-sharing demand prediction. In stations with similar geographical environment, people’s travel times and destinations exhibit similarities. Hence, analyzing the similarity of the geographical environment between stations helps to identify stations with common characteristics in the spatial dimension, thereby enhancing the model’s ability to capture stochastic features. Moreover, as the value of Pearson Correlation Coefficient approaches 1, the positive correlation between the two station temporal sequences increases, indicating that their usage patterns are more similar in the spatial dimension. Therefore, we have measured the spatial similarity usage patterns between stations by calculating the Pearson coefficient of the station geographic characteristics between stations. The specific formula is as follows:

$$\begin{array}{c} r(X,Y)=\frac{\sum_{i=1}^{n}\left(X_{i}-\bar{X})(Y_{i}-\bar{Y}\right)}{\sqrt{\sum_{i=1}^{n}{\left(X_{i}-\bar{X}\right)}^{2}}\sqrt{\sum_{i=1}^{n}{\left(Y_{i}-\bar{Y}\right)}^{2}}} \end{array}$$ (22)

where *X*_*i*_ and *Y*_*i*_ denote the *i*^*th*^ station geographic characteristics, respectively. $\bar{X}$ and $\bar{Y}$ denote the means of station geographic characteristics. Fig 6 displays a heatmap illustrating the spatial similarity between stations.

#### Spatio-temporal similarity calculator

**Fig 7:**
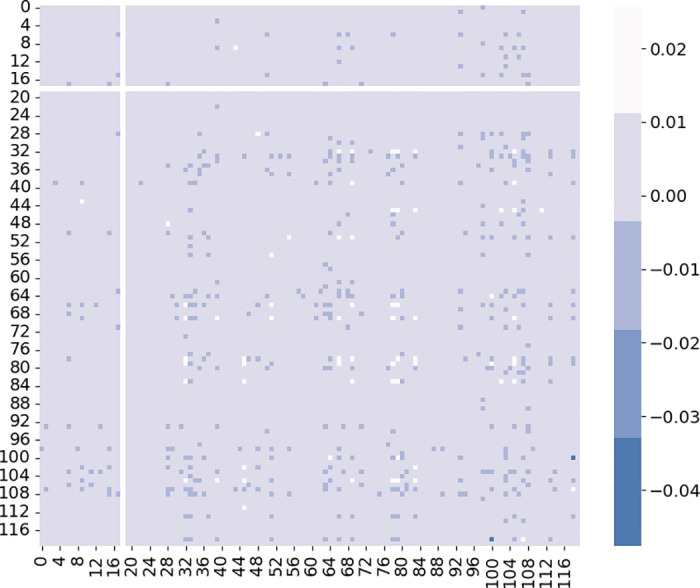
Spatio-temporal similarity between stations.

Taking into account the influence of temporal-spatial factors on stations, we use spatio-temporal composite metrics to measure the similarity of bike usage patterns between stations. The similarity usage pattern features are depicted in Eq 24.

$$\begin{array}{c} V=(\begin{array}{ccc} \infty & \ldots & v(s_{1},s_{n})\\ \vdots & \ddots & \vdots \\ v(s_{n},s_{1}) & \cdots & \infty \end{array}) \end{array}$$ (23)

$$\begin{array}{c} v(s_{i},s_{j})=\omega_{1}tsv(s_{i},s_{j})+\omega_{2}r(s_{i},s_{j}) \end{array}$$ (24)

where *v* denotes the matrix of similarity of spatio-temporal usage patterns of stations. *ω*_1_ and *ω*_2_ are learnable parameters. *tsv*(*s*_*i*_, *s*_*j*_) denotes the similarity values of usage patterns between stations *s*_*i*_ and *s*_*j*_ in the temporal dimension. And *r*(*s*_*i*_, *s*_*j*_) denotes the similarity values of usage patterns between stations *s*_*i*_ and *s*_*j*_ in the spatial dimension. Fig 7 displays a heatmap illustrating the spatio-temporal similarity between stations.

### Bike-sharing demand prediction

**Fig 8:**
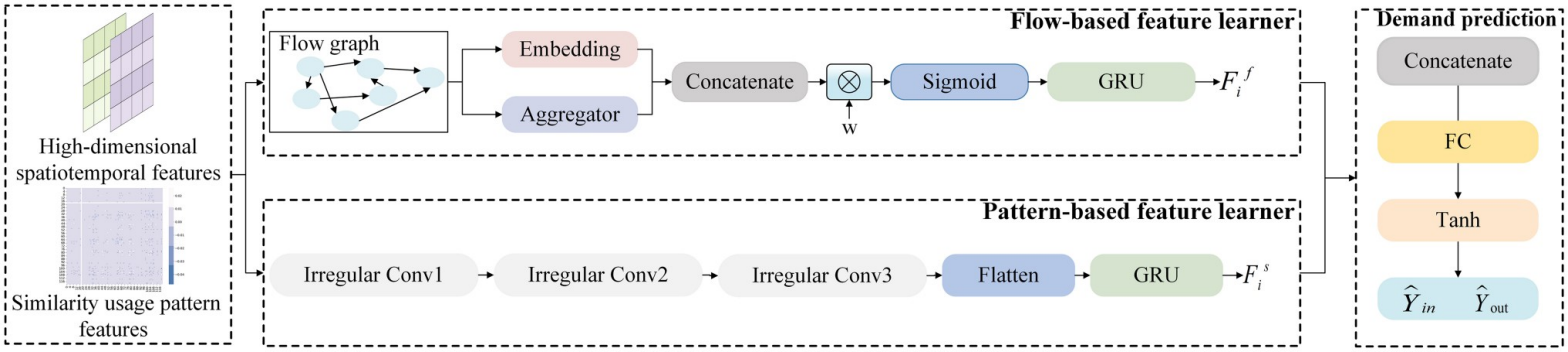
Bike-sharing demand prediction module.

In complex urban public transportation systems, bike-sharing demand exhibits a complex spatio-temporal correlation. Traditional bike-sharing demand prediction learns the connections of stations by aggregating information from their neighboring stations. However, the borrowing and returning behavior of users between stations establishes a hidden correlation between them, which conveys more information than the connections between neighboring stations. By aggregating information from this correlation to learn inter-station features, prediction performance can be significantly improved. Furthermore, the usage of bike-sharing at neighboring stations can exhibit considerable variations due to temporal fluctuations in travel behavior and the distinct characteristics of the built environment. On the other hand, even for stations that are geographically distant from one another, their bike-sharing usage patterns may exhibit remarkable similarity. If the correlations between similar stations can be accurately captured and effectively incorporated into the prediction model, it may significantly improve the accuracy and reliability of bike-sharing demand prediction. Thus, we employ a dual network structure to learn the hidden correlated features among stations based on their flow and usage patterns similarity. In Fig 8, we present the bike-sharing demand prediction module, which consists of two key components: flow-based feature learner and pattern-based feature learner. Then, the extracted features are utilized by the bike-sharing demand prediction to realize the final prediction.

#### Flow-based feature learner

The borrowing and returning behaviors of users establish flow connections between stations. The greater the flow between stations, the stronger their interdependence. By aggregating the characteristics of stations with strong correlations through flow relationships, we can avoid introducing weak correlations, thus improving the performance and efficiency of prediction. However, existing conventional aggregation functions might not be suitable for capturing the flow characteristics of bike-sharing data. Therefore, we propose the FFL, which is designed to extract the spatio-temporal correlations between bike-sharing stations by analyzing the flow of bikes between them.

In the beginning, we use $\hat{I}$ and $\hat{O}$ to construct flow graph. Specifically, at time *t*, it is expressed as *G*_*t*_ = (*N*_*t*_, *E*_*t*_). The node of graph is represented as $N_{i}^{t}=(s_{i},{\text{T}}_{i}^{t})$ where $T_{i}^{t}$ is the feature of station *s*_*i*_ at time *t*. The edge of between *s*_*i*_ and *s*_*j*_ is $E_{i,j}^{t}=(e_{i,j}^{t},w_{i,j}^{t})$. When $|{\hat{I}}_{i,j}-{\hat{O}}_{i,j}|>0$, *e*_*i*,*j*_ = 1, conversely *e*_*i*,*j*_ = 0. And the weight between *s*_*i*_ and *s*_*j*_ at time *t* is defined as $w_{i,j}^{t}$:

$$\begin{array}{c} w_{i,u}=\frac{{\text{T}}_{i,u}^{t}}{\sum_{k\in S}{\text{T}}_{i,k}^{t}} \end{array}$$ (25)

where *S* is the number of bike station.

Then, we develop a flow aggregator to improve the GNN. Specifically, $F^{0}=\{F_{1}^{0},F_{2}^{0},\ldots ,F_{i}^{0},\ldots ,F_{n}^{0}\}$ is the initial high-dimensional spatio-temporal features, where $F_{i}^{0}={\text{T}}_{i}$. And ${\text{T}}_{i}$ is shown in Eq 15. By utilizing the high-dimensional spatio-temporal features of stations that are highly correlated with station *s*_*i*_ in terms of flow, we can update the high-dimensional spatio-temporal features $F_{i}^{k}$ of station *s*_*i*_:

$$\begin{array}{c} F_{i}^{k}=\sigma (W^{k}\cdot Aggr(\left\{F_{i}^{k-1}\}\cup \{F_{j}^{k-1},\forall s_{j}\in ℵ(s_{i})\right\})) \end{array}$$ (26)

where ℵ(*s*_*i*_) is the neighboring stations of *s*_*i*_ in the graph. *W*^*k*^ is learnable parameter. And *Aggr*(*) is the flow aggregator in our network which aggregate the high-dimensional spatio-temporal features from one’s neighboring nodes. It is computed by:

$$\begin{array}{c} Aggr(\left\{F_{i}^{k-1}\}\cup \{F_{j}^{k-1},\forall s_{j}\in ℵ(s_{i})\right\})=\sum w_{i,u}F_{u}^{k-1} \end{array}$$ (27)

where $F_{u}^{k-1}\in \{F_{i}^{k-1}\}\cup \{F_{j}^{k-1},\forall s_{j}\in ℵ(s_{i})\}$. And in Eq 25, we provide the calculation method for *w*_*i*,*u*_.

Extracting temporal dependency in the graph using GRU. We $F_{i}^{f}$ is used to show the final embedding of station *s*_*i*_ in the flow-convoluted graph.

#### Pattern-based feature learner

Traditional prediction models often assume that neighboring stations are highly correlated. However, with temporal variations in user behavior and geographic characteristics of bike-sharing stations, the demand for bike-sharing at neighboring stations can vary significantly. On other hand, the non-neighboring stations exhibit greater similarity in their temporal and spatial usage patterns than neighboring stations. Aggregating the information of stations with similar usage patterns is more conducive to improving the accuracy of bike-sharing demand prediction models. Consequently, we develop a pattern-based feature learner to learn the dependency of bike usage among similar usage pattern stations.

**Fig 9:**
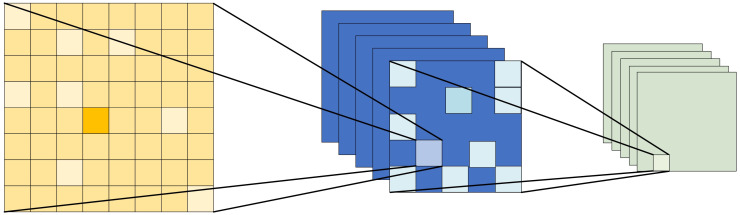
Irregular convolution.

The pattern-based feature learner adopts a multi-layer irregular convolutional architecture to capture the characteristics of bike-sharing demand among stations based on the similarity usage pattern features. The output of the irregular convolution is fed into a GRU. The aim is to extract the temporal correlation in bike-sharing demand. In this case, the irregular convolutional network structure is shown in Fig 9.

For each central station in the network, we identify the top *k* − 1 stations based on similarity usage pattern features, which then undergo convolution with the stations. This involves irregular convolutional computation:

$$\begin{array}{c} F_{i,j}=\sum (b_{i,u}+\sum_{c=1}^{C_{in}}\sum_{s=1}^{S}N_{c}^{s}(i,u)w_{c}^{s}) \end{array}$$ (28)

where *C*_*in*_ denotes the number of channels in input *T*. *S* denotes the number of convolutional kernels. $N_{c}^{s}(i,u)$ denotes the neighbors with similar bike usage patterns to the central unit *s*_*i*_ in channel *c*. $w_{c}^{s}$ denotes the weight in the convolutional kernel corresponding to the neighbor $N_{c}^{s}(i,u)$. *b*_*i*, *u*_ is the learnable parameter. The $F_{i}^{s}$ use to denote the final embedding of station *s*_*i*_ in the network.

#### Demand prediction

To put it simply, this aims to consider both the impact of bike flow and bike usage pattern similarity on a station. We concatenate $F_{i}^{f}$ and $F_{i}^{s}$:

$$\begin{array}{c} F_{i}=F_{i}^{f}∥F_{i}^{s} \end{array}$$ (29)

where ∥ is the concatenating operation. And *F*_*i*_ is the finally embedding of station *s*_*i*_. And then, we feed the embedding *F*_*i*_ of station *s*_*i*_ to a FC layer for predicting the demand and supply of individual station at time *t*:

$$\begin{array}{c} ({\hat{x}}_{i}^{t},{\hat{y}}_{i}^{t})=\sum_{\text{i}=1}^{N}F_{i}^{t}\cdot \omega_{\text{i}}^{t} \end{array}$$ (30)

where ${\hat{x}}_{i}^{t}$ and ${\hat{y}}_{i}^{t}$ are the prediction results of station *s*_*i*_ demand and supply at time *t*, respectively. And $\omega_{\text{i}}^{t}$ is learnable parameter.

## Data description and benchmark models

### Dataset

In subsequent experiments, we will use real-world datasets: BikeNY (bike order records collected by New York City) and POINY (points of interest in New York City). The details of these two datasets are as follows:

BikeNY: It contains daily bike-sharing trip records for 120 stations from July 1, 2013 to February 28, 2014 in New York City. Each order record mainly consists of the pick-up or drop-off start time, pick-up or drop-off end time, station name, station longitude, station latitude, and other information. Data collected from July 1 to December 17 as the training set, data collected from December 18 to January 11, 2014 as the validation set, and data collected from January 12 to February 28 as the test set.

POINY: It contains valuable information about various points of interest, such as their categorization, longitude and latitude, as well as their respective names, from the New York City government.

### Experiment setup

We build the proposed model on PyTorch, supported by the Python library. Concurrently to prevent the model from being overly focused on variations in features during its learning process, which may lead to inaccurate feature representation and the emergence of the gradient explosion issue, we utilize the z-score [39] technique to normalize the single-vehicle data constructed for experimentation, as depicted in Eq 31.

$$\begin{array}{c} x^{\prime }=\frac{x-\mu }{\sigma } \end{array}$$ (31)

where *μ* denotes the mean. And *σ* denotes the standard deviation.

For the other hyperparameters in the model, we set the time interval to 15 minutes or 30 minutes and the length of daily cycle to 96 or 48. As for the time dimensions of the input short-term inflow/outflow matrix and long-term inflow/outflow matrix, we set c to 12 (representing 12 consecutive time intervals) and d to 7 (representing a continuous 7 days). Furthermore, as the user usage patterns of the stations are influenced by the building environment within a 150-meter radius, we use the count of 8 POI entity classes within a 150-meter radius of each station to construct the geographical features of the stations.

### Evaluation measurement

Moreover, in model comparison, two commonly used indicators were used to evaluate the predictive performance of bike-sharing demand: Mean Squared Error (MSE) and Mean Absolute Error (MAE). These two indicators are widely used in the field of predictive modeling to measure the accuracy of predictions. MSE measures the average squared difference between the predicted and actual values, while MAE measures the average absolute difference between the predicted and actual values. Both indicators provide valuable information about the performance of a predictive model. Their equations are as follows:

$$\begin{array}{c} MSE=\frac{\sum_{i=1}^{N}{\left(y_{in}^{i}-{\hat{y}}_{in}^{i}\right)}^{2}+\sum_{i=1}^{N}{\left(y_{out}^{i}-{\hat{y}}_{out}^{i}\right)}^{2}}{2N} \end{array}$$ (32)

$$\begin{array}{c} MAE=\frac{\sum_{i=1}^{N}\left(y_{in}^{i}-{\hat{y}}_{in}^{i})+\sum_{i=1}^{N}(y_{out}^{i}-{\hat{y}}_{out}^{i}\right)}{2N} \end{array}$$ (33)

where *N* denotes the number of stations. ${\hat{y}}_{in}^{i}$ and ${\hat{y}}_{out}^{i}$ denote the predictable demand and supply. $y_{in}^{i}$ and $y_{out}^{i}$ denote the actual value of demand and supply.

### Benchmark models

Five benchmark models are adopted for performance comparison with FF-STGCN, including one Time series model (LSTM) and four Graph Convolutional models (STGCN, MSTGCN, STSGCN and MC_STGCN):

LSTM: LSTM can capture the temporal dependency for both short-term and long-term of time by introducing gate theory [26].

MC_STGCN: Its adepts a graph convolution network, based on the Louvain algorithm, effectively captures the regional spatio-temporal dependency [8].

STSGCN: It uses a synchronous graph convolution network to capture the spatio-temporal dependency at the complex local environments [40].

ST-GCN: It is combines graph convolutional networks and temporal convolutional networks to analyze spatio-temporal data [41].

MSTGCN: It adeptly embodies the intricate spatio-temporal characteristics of the data by leveraging non-Euclidean spatial graphs. And it captures spatio-temporal dependency by multi-graph convolution and context-gated recurrent neural networks [42].

## Experimental results and discussion

### Performance comparison

We present a comparison of the overall accuracy achieved by our proposed model with that of several baseline models. Table 1 shows a comparison of the predicted errors of benchmark models and our model for predicting bike-sharing demand on the BikeNY dataset.

**Table 1:** Performance comparison across models.

Model Name	*l*_*c*_ = 15min		*l*_*c*_ = 30min	
	MSE	MAE	MSE	MAE
LSTM	0.38455	0.39866	0.465384	0.51166
ST-GCN	0.35333	0.32535	0.34429	0.35435
MSTGCN	0.35274	0.3211	0.3440	0.3142
STSGCN	0.3683	0.3247	0.3441	0.3247
MC_STGCN	0.35823	0.34099	0.3034	0.3012
**FF-STGCN**	**0.27448**	**0.29621**	**0.23636**	**0.293317**

Generally, our proposed model consistently outperforms other benchmark models when it comes to prediction accuracy across the 15-minute and 30-minute time slots in the BikeNY dataset. Notably, our proposed model achieves a significant reduction of MSE and MAE by 31.29% and 9.53%, compared to the model with the outperformance in five benchmark models at 30-minute time slot. Furthermore, within a 15-minute time slot, compared to the best-performing model among the five benchmark models, our proposed model significantly reduced MSE and MAE by 9.72% and 2.58%, respectively.

The results indicate that the hybrid model that couples GCN and LSTM model to learn features of bike-sharing demand outperforms LSTM in all time intervals. This is due to the fact that LSTM is a typical time series approach and it cannot exploit the spatial dependency of bike-sharing demand among stations for prediction. And it illustrates that capturing the spatial dependencies between stations facilitates improved prediction performance.

STSGCN has further improvement than ST-GCN. This result could be due to the fact that STSGCN captures heterogeneity in local spatio-temporal maps. MSTGCN has a considerable improvement over STSGCN which demonstrates the importance of considering the usage pattern similarity among stations and the effectiveness of multi-structural network for capturing the spatial correlation. Nevertheless, MSTGCN is inclined to focus on dependency from neighboring stations, it cannot sufficiently consider the correlation on distant stations which have similar usage pattern. Consequently, although MSTGCN achieves good prediction results, the performance of our proposed model is still better than it in all indicators.

The prediction accuracy of our model is much higher than MC_STGCN across two time slots. This is because, compared to MC_STGCN, our proposed model encodes the long-term correlation through the fusion of short-term and long-term flow features, rather than combining them into a singular feature vector. Such results imply that the fusion of short-term and long-term traffic features is more effective than combining them for predicting bike-sharing usage.

Our proposed model designs network structures that are specific to different characterisations, allowing for more effective learning compared to using a single network structure. Moreover, we replace the spatial neighbors with semantic neighbors in irregular convolutions. Thus, the results of compared with MSTGCN and STSGCN, among 30-minute time slot, our approach achieves an improvement of MSE and MAE by 31.29% and 39.39%.

**Fig 10:**
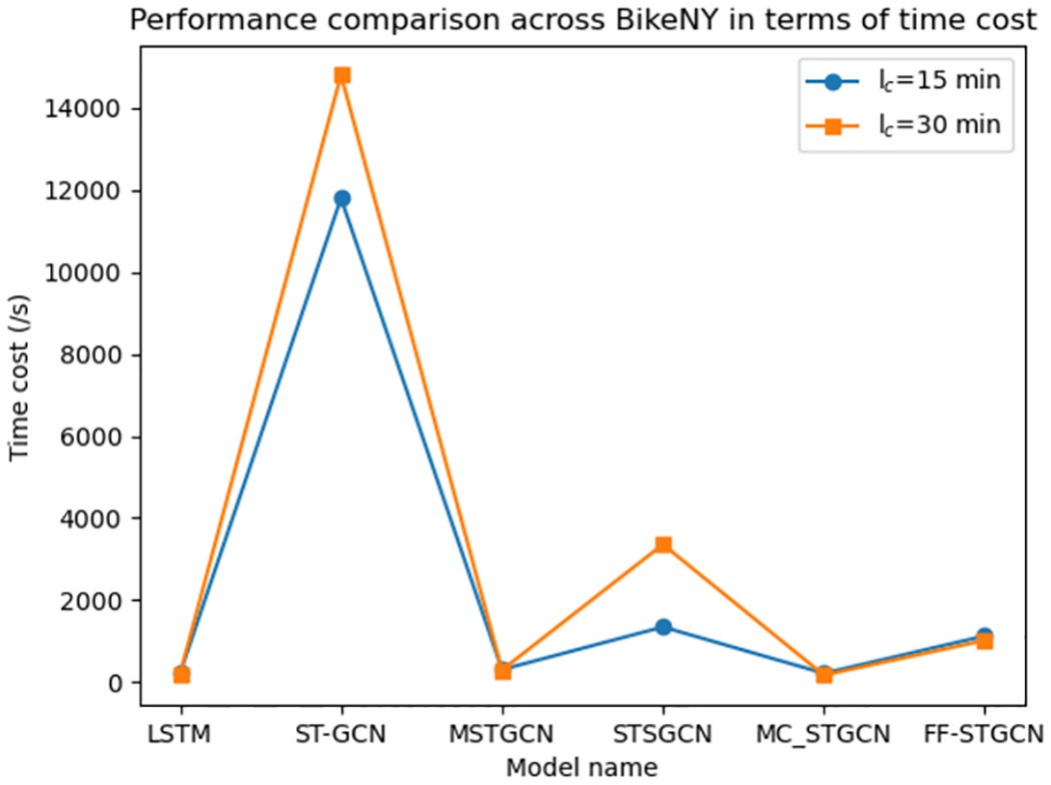
Performance comparison across models in terms of time cost.

However, as shown in Fig 10, our proposed model incurs higher time costs compared to time series model LSTM and certain graph convolutional networks (such as MSTGCN, MC_STGCN). In contrast to the LSTM model, our model introduces a network module designed to capture spatial dependencies, giving it a more complex network structure that requires additional time for parameter optimization during training. Meanwhile, unlike our model, which focuses on predicting the supply and demand of bike-sharing at individual stations, the MC_STGCN model only needs to predicate the overall demand for bike-sharing within a specific region, reducing data volume and computational complexity. Compared to the MSTGCN model, our proposed model considers both the spatio-temporal features of bike-sharing usage patterns between stations and the spatio-temporal features of traffic between stations, resulting in an overall more complex network architecture and longer training time. Additionally, our model introduces a module for learning the similarity of user usage patterns, dynamically capturing the similarity between usage patterns at different stations. The use of the DTW algorithm and Pearson coefficient calculation in this module, however, increases the time complexity and computational costs of our model.

### Performance of models at stations with different bike usage levels

Satisfying users’ travel needs is one of the important tasks in the bike-sharing system. However, the bike-sharing demand is not evenly distributed in urban areas which results in a discrepancy in the quantity of bikes present at various demand stations. Therefore, if the demand and supply in stations with different usage patterns can be precisely predicted, it is helpful for the bike-sharing rebalancing problem.

We categorize the stations into four levels based on the hourly bike usage in the New York City bike-sharing system to evaluate the models’ performance across these levels within the 30-minute time slot [37]. Specifically, stations with an hourly demand in the range (106, 141] are classified as high-demand level (grade 1); those with demand in the range (85, 106] are considered as moderately high-demand level (grade 2); those with demand in the range (59, 85] fall into the moderately low-demand level (grade 3); and stations with demand in the range (0, 59] are labeled as low-demand level (grade 4).

**Fig 11:**
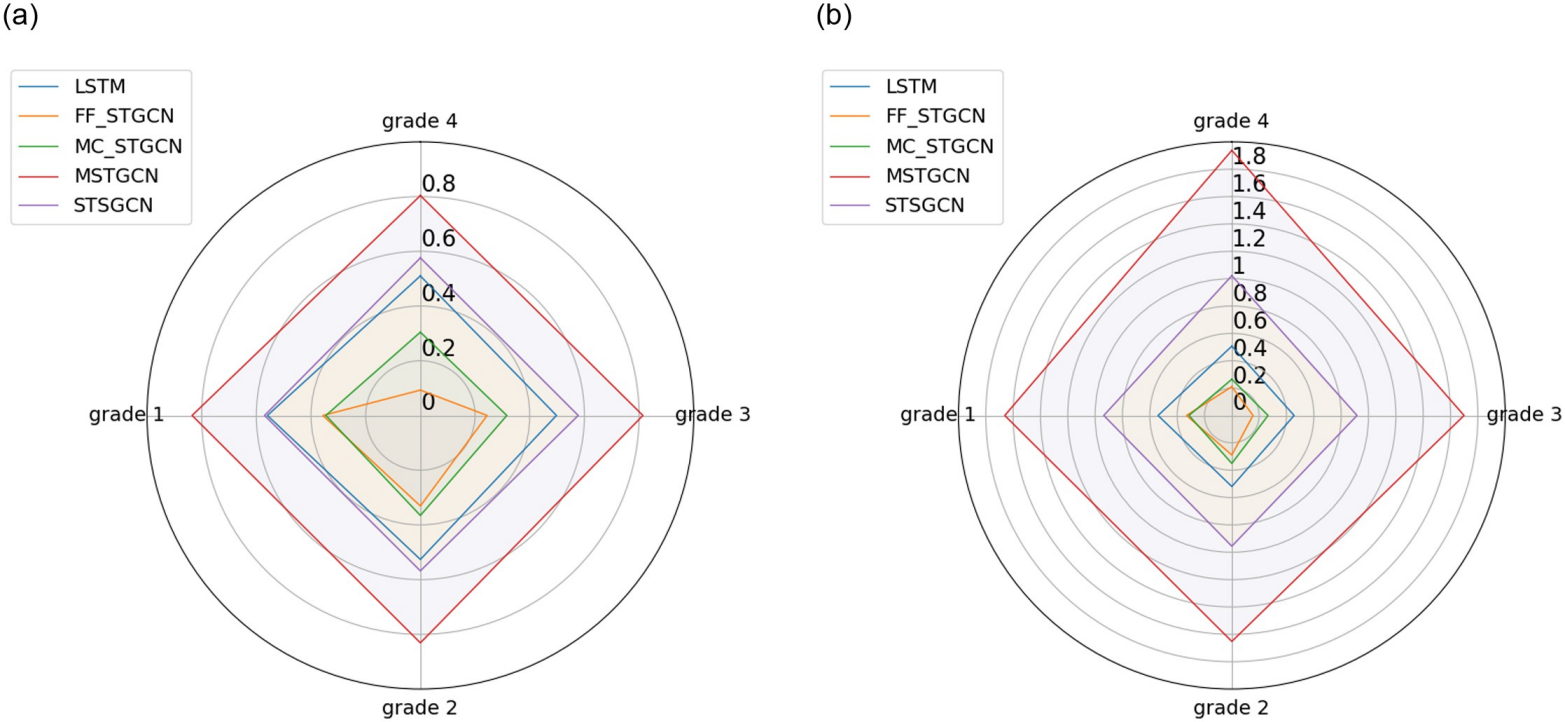
Performance of models at stations with different bike usage levels. (a) MAE (b) MSE.

Fig 11 shows the MAE and MSE distributions of each quantile of bike usage. Experimental results demonstrate that our model outperforms four benchmark models at stations with different bike usage levels. In particular, at stations with low demand, the predicted error of our proposed model is lower than other benchmark models. However, at stations with high demand, the performance of our proposed model is similar to that of LSTM. In addition, our proposed model still outperforms other benchmark models at other orders of magnitude of bike usage rates. Overall, FF-STGCN achieves better performance at stations with different bike usage levels.

### Performance of models at stations during peak hours

Satisfying users’ travel needs during morning and evening peak hours is important. This is because many users choose to ride bikes during these time periods to solve the last mile problem. Therefore, accurate prediction of bike-sharing demand can help operators develop appropriate rebalancing options to meet subscriber needs at the minimum cost. For this reason, in our experiment, we predict the supply and demand of bike-sharing during morning peak hours (from 6:30 am to 10:00 am) and evening peak hours (from 5:00 pm to 8:00 pm) separately. We use MSE and MAE to evaluate model performance.

**Fig 12:**
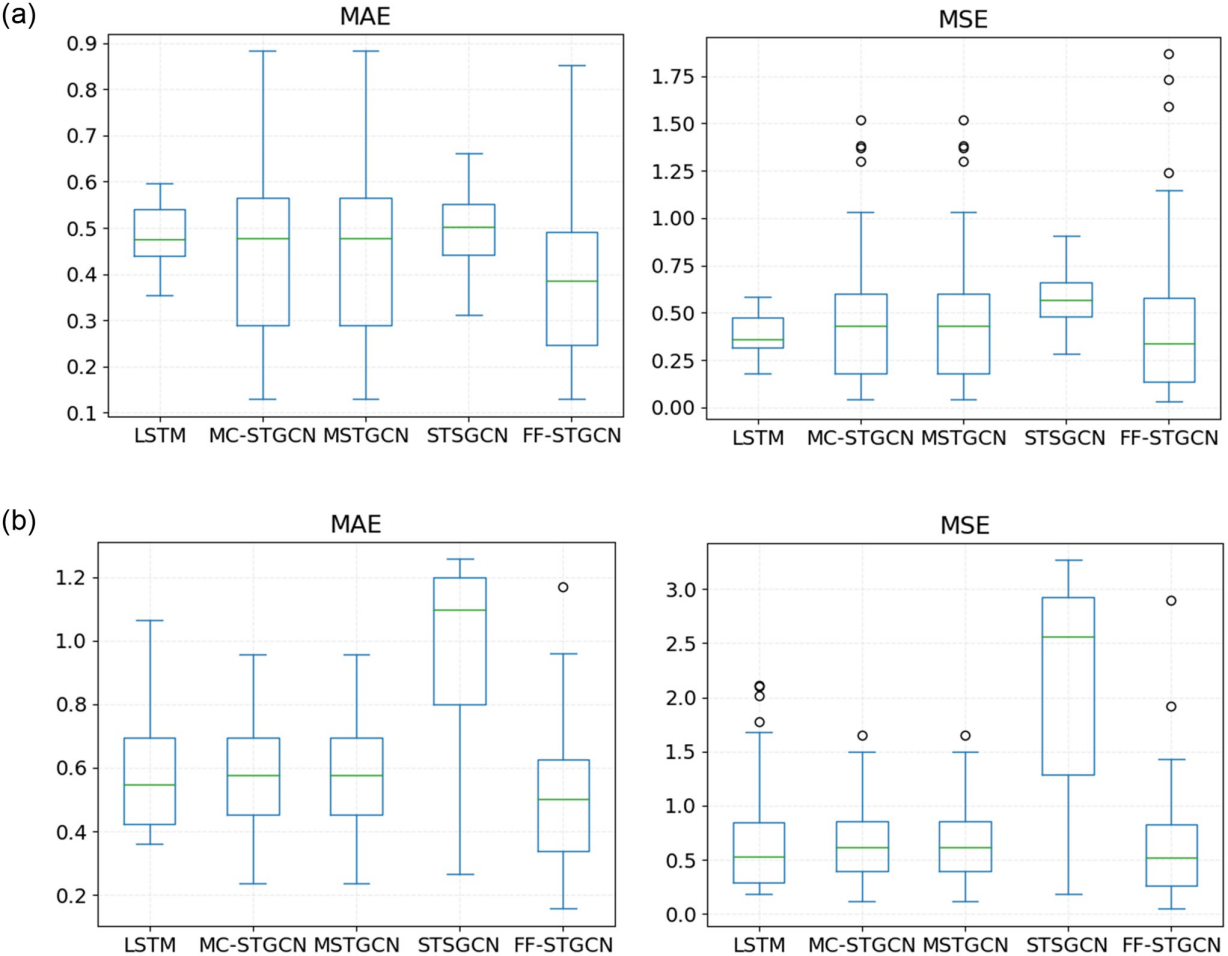
Performance of models during peak hours. (a) Morning peak (6:30-10:00AM) (b) Evening peak (5:00-8:00PM).

Fig 12 represents a comparison of the predictive performance of various models during peak periods. The pilot results show that the average predicted error of our proposed model is the lowest during both peak hours. Specifically, during the morning peak, the average MAE of FF-STGCN is smaller than that of other benchmark models. In addition, both the MAE and MSE of FF-STGCN are better than those of the benchmarks during the evening peak. This indirectly proves that our proposed model outperforms the benchmark models during peak periods.

### Ablation study

#### Comparative analysis spatio-temporal module

**Fig 13:**
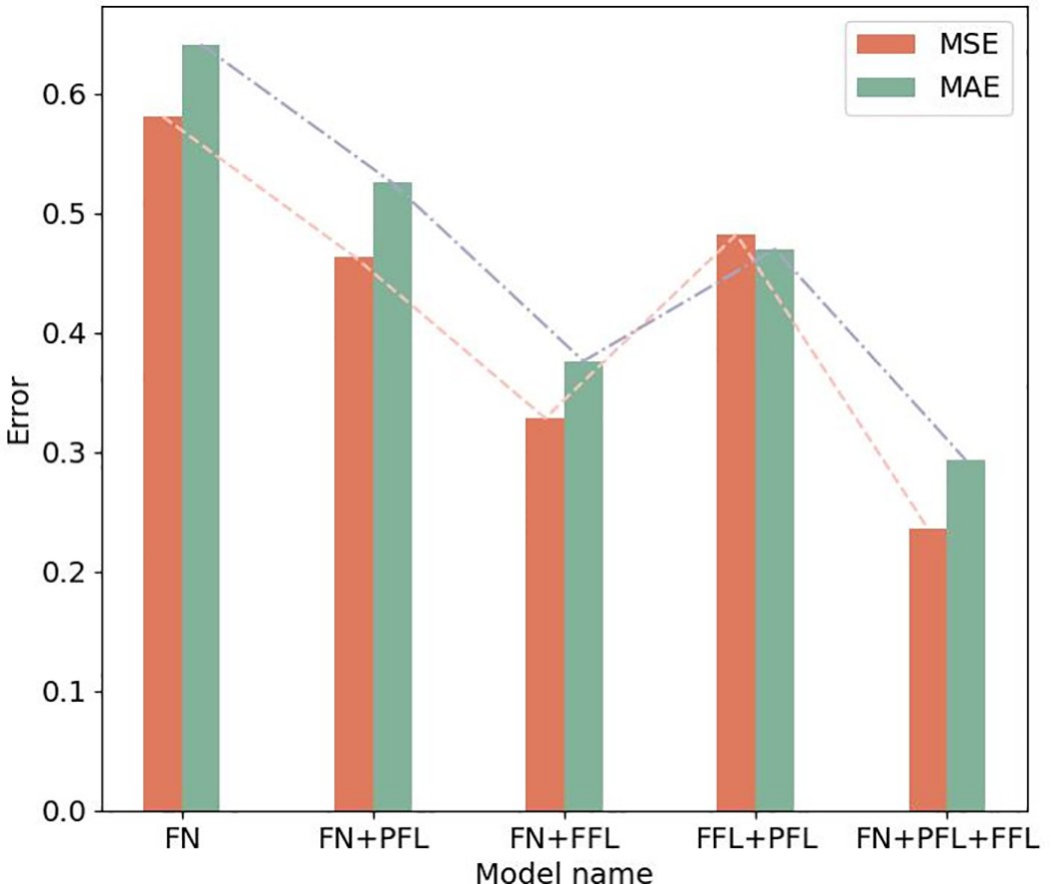
Comparative spatio-temporal module.

To validate the effect of different spatio-temporal modules on our model, various combinations of modules were fixed, namely the multi-scale spatio-temporal feature fusion module (FN), the flow-based feature learner (FFL), and the pattern-based feature learner (PFL). The results can be found in Table 2. And a visual representation can be seen in Fig 13.

**Table 2:** Comparative spatio-temporal module.

Combinations of module	MSE	MAE
FN	0.58105	0.64137
FN+PFL	0.46401	0.525622
FN+FFL	0.32845	0.3763
FFL+PFL	0.48233	0.470021
**FN+FFL+PFL**	**0.23636**	**0.293317**

The analysis of the experimental results can be concluded as follows:

- Each spatio-temporal module contributes to building accurately prediction results. As the combination of modules increases, the predicted error decreases. This result indicates that models considering multiple scales and features are superior to models considering only a single feature or scale.
- The combination model of FN+PFL or FN+FFL shows improvement over the model of single module FN. The consideration of flow or similarity bike usage patterns features were both beneficial in improving the prediction of bike-sharing demand.
- The combination model of FF+FFL outperforms FF+PFL. The experimental results indicate that bike-sharing demand and supply prediction is more sensitive to flow features than to similarity bike usage patterns features.
- When comparing FN+FFL+PFL model with FFL+PFL modules, the FN+FFL+PFL model showed better prediction performance. This demonstrates that incorporating multi-scale temporal features is beneficial in improving prediction results.

#### Comparative analysis metrics of bike usage patterns similarity

To delve into understanding the metrics for quantifying the similarity of bike usage patterns between stations, the performance of FF-STGCN and two variants of FF-STGCN is evaluated in this study. The two variants are: one that utilizes DTW to quantify the similarity of bike usage patterns in the temporal dimension and another that incorporates the Pearson coefficient to quantify the similarity of bike usage patterns in the spatial dimension. We have named them FF-STGCN:P and FF-STGCN:D, respectively. In order to ensure the accuracy of our experimental results, we set the hyperparameters of the two variant models identical to those of the original model.

Table 3 represents the performance of two variants of FF-STGCN and FF-STGCN in 30-minute time slots based on two indicators (MAE and MSE). We find that the prediction error of the variants with the Pearson measure is lower than the variants with the DTW metric. The results indicate that for bike usage patterns, metrics based on the spatial dimension can more accurately quantify the similarity than those based on the temporal dimension. However.FF-STGCN:P achieves good prediction results, the performance of our proposed model is still better than it in all indicators. This further demonstrates that considering the similarity of bike usage patterns between stations based on both temporal and spatial dimensions can help improve the accuracy of bike-sharing demand prediction.

**Table 3:** Comparative analysis metrics of similar bike usage patterns.

Model Name	MSE	MAE
FF-STGCN:D	0.3214	0.5782
FF-STGCN:P	0.315411	0.488125
**FF-STGCN**	**0.23636**	**0.293317**

**Fig 14:**
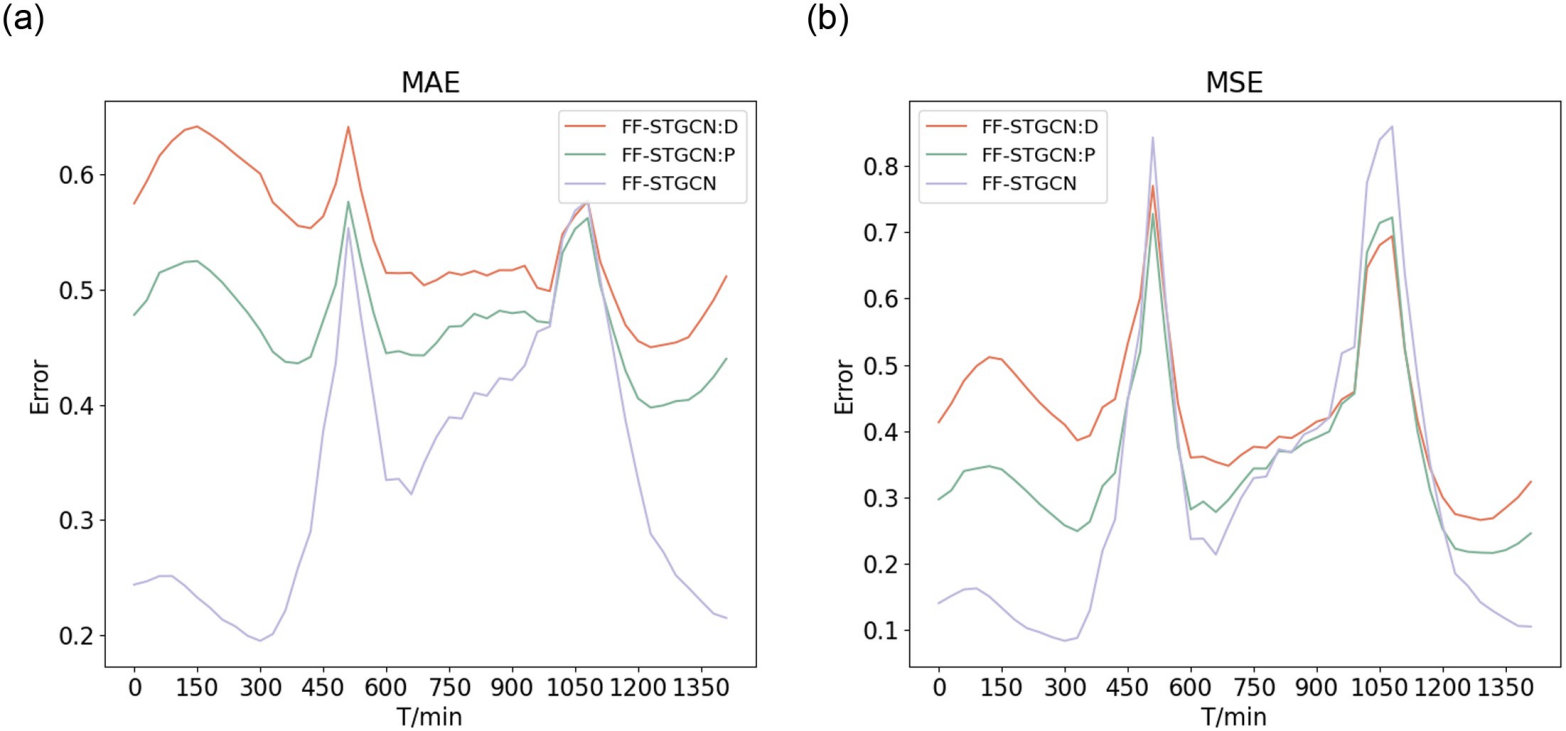
Comparative metrics of bike usage patterns similarity. (a) MAE (b) MSE.

Fig 14 demonstrates the prediction error of two variants of FF-STGCN and FF-STGCN during the overall day. Compared with the two variants of FF-STGCN and FF-STGCN, our proposed model has better performance during daytime hours. The results show that our model has better prediction performance in time periods with high usage. In addition, it is shown that the spatio-temporal composite metric is an efficient method for selecting semantic neighborhoods involved in irregular convolution.

## Conclusion

In this paper, we propose a usage pattern similarity based dual-network for bike-sharing demand prediction, called FF-STGCN, and evaluate it on BikeNY dataset. We compare FF-STGCN with five benchmark models to verify its effectiveness in a real data environment. Based on the results of prediction, our model universally outperforms the five benchmark models across two time periods. Meanwhile, we perform a comparative analysis with benchmark models on different stations and peak hours with varying levels of bike use. The result of the predicted error reflects that our model has lower errors than other benchmark models in stations. Furthermore, we design ablation studies to assess the contribution of each component in our model, namely the multi-scale spatio-temporal feature fusion module, the bike usage pattern similarity learning module, and the bike-sharing demand prediction module. The results of ablation studies show that flow features are more influential in prediction accuracy than similar bike usage patterns feature, and the model with consideration of similar bike usage patterns yields more accurate prediction results than the model with only learning features based on spatial neighbors.

The contribution of this research can be mainly summarized in the following three aspects: (1) We develop a multi-scale spatio-temporal feature fusion module to address limitations in multi-scale spatio-temporal accuracy. The feature training uses a MS-FA network to train long-term and short-term features separately, capturing the characteristics of bike-sharing across various time slots. Then, through feature fusion, the short-term and long-term features of bike-sharing demand are integrated to provide more comprehensive and in-depth spatio-temporal feature for subsequent feature learners. (2) We construct a bike usage pattern similarity learning module to extract hidden correlated features among stations. By doing so, it can effectively reduce the influence of random fluctuations of individual stations on feature extraction, enrich the features, enhance the stability of the model, and improve prediction accuracy. (3) We have designed a bike-sharing demand prediction model that employs a dual network structure containing flow-based feature learner and pattern-based feature learner. By the dual network structure to capture feature dependency. Then, the output results of the two feature learners are comprehensively considered as the final prediction result. Consequently, the FF-STGCN model, which considers multi-scale features and multiple features, can prevent the model from being affected by time-lagged demand and supply-demand imbalances across stations. It shows potential and promising capacity in bike-sharing demand prediction.

However, our research has limitations when it comes to complex bike-sharing systems. Therefore, our future work includes the following aspects: (1) Because bike-sharing demand is affected by various external factors such as sudden events and weather, we will analyze and introduce these external factors to predict bike-sharing demand in extreme environments. (2) We will consider more information about the users themselves, such as age and occupation, to analyze the riding habits of different user groups and improve the accuracy of the estimation results of the similarity of bike usage patterns at stations. (3) We will use a bike-sharing demand prediction model and find the optimal path between stations to save dispatching costs.

## References

[1] Jiang Weiwei. Bike sharing usage prediction with deep learning: a survey. Neural Computing and Applications, 2022, 34(18): 15369–15385 doi: 10.1007/s00521-022-07380-5 35702665 PMC9185130

[2] Tomaras Dimitrios and Boutsis Ioannis and Kalogeraki Vana. A holistic approach for modeling and predicting bike demand. Information Systems, 2023, 111: 102129. doi: 10.1016/j.is.2022.102129

[3] Eren E, Uz V E. A review on bike-sharing: The factors affecting bike-sharing demand. Sustainable cities and society, 2020, 54: 101882. doi: 10.1016/j.scs.2019.101882

[4] Kim K. Spatial contiguity-constrained hierarchical clustering for traffic prediction in bike sharing systems. IEEE Transactions on Intelligent Transportation Systems, 2021, 23(6): 5754–5764. doi: 10.1109/TITS.2021.3057596

[5] Du Y, Deng F, Liao F. A model framework for discovering the spatio-temporal usage patterns of public free-floating bike-sharing system. Transportation Research Part C: Emerging Technologies, 2019, 103: 39–55. doi: 10.1016/j.trc.2019.04.006

[6] Almannaa M H, Elhenawy M, Rakha H A. A novel supervised clustering algorithm for transportation system applications. IEEE transactions on intelligent transportation systems, 2019, 21(1): 222–232. doi: 10.1109/TITS.2018.2890588

[7] Song J, Zhang L, Qin Z, et al. A spatiotemporal dynamic analyses approach for dockless bike-share system. Computers, Environment and Urban Systems, 2021, 85: 101566. doi: 10.1016/j.compenvurbsys.2020.101566

[8] Tang J, Liang J, Liu F, et al. Multi-community passenger demand prediction at region level based on spatio-temporal graph convolutional network. Transportation Research Part C: Emerging Technologies, 2021, 124: 102951. doi: 10.1016/j.trc.2020.102951

[9] Caggiani L, Camporeale R, Ottomanelli M, et al. A modeling framework for the dynamic management of free-floating bike-sharing systems. Transportation Research Part C: Emerging Technologies, 2018, 87: 159–182. doi: 10.1016/j.trc.2018.01.001

[10] Wang Y J, Kuo Y H, Huang G Q, et al. Dynamic demand-driven bike station clustering. Transportation Research Part E: Logistics and Transportation Review, 2022, 160: 102656. doi: 10.1016/j.tre.2022.102656

[11] Wang B, Tan Y, Jia W. TL-FCM: A hierarchical prediction model based on two-level fuzzy c-means clustering for bike-sharing system. Applied Intelligence, 2022: 1–18.

[12] Gu J, Zhou Q, Yang J, et al. Exploiting interpretable patterns for flow prediction in dockless bike sharing systems. IEEE Transactions on Knowledge and Data Engineering, 2020, 34(2): 640–652. doi: 10.1109/TKDE.2020.2988008

[13] Zhao S, Zhao K, Xia Y, et al. Hyper-clustering enhanced spatio-temporal deep learning for traffic and demand prediction in bike-sharing systems. Information Sciences, 2022, 612: 626–637. doi: 10.1016/j.ins.2022.07.054

[14] Harikrishnakumar R, Nannapaneni S. Forecasting Bike Sharing Demand Using Quantum Bayesian Network. Expert Systems with Applications, 2023, 221: 119749. doi: 10.1016/j.eswa.2023.119749

[15] Leem S, Oh J, Moon J, et al. Enhancing multistep-ahead bike-sharing demand prediction with a two-stage online learning-based time-series model: insight from Seoul. The Journal of Supercomputing, 2023: 1–34.

[16] Hernandez-Matamoros A, Fujita H, Hayashi T, et al. Forecasting of COVID19 per regions using ARIMA models and polynomial functions. Applied soft computing, 2020, 96: 106610. doi: 10.1016/j.asoc.2020.106610 32834798 PMC7409837

[17] Kumar K, Jain V K. Autoregressive integrated moving averages (ARIMA) modelling of a traffic noise time series. Applied Acoustics, 1999, 58(3): 283–294. doi: 10.1016/S0003-682X(98)00078-4

[18] Avuglah R K, Adu-Poku K A, Harris E. Application of ARIMA models to road traffic accident cases in Ghana. International journal of statistics and applications, 2014, 4(5): 233–239.

[19] Cortez-Ordoñez A, Vázquez P P, Sanchez-Espigares J A. Scalability evaluation of forecasting methods applied to bicycle sharing systems. Heliyon, 2023, 9(10). doi: 10.1016/j.heliyon.2023.e20129 37810852 PMC10556600

[20] Ma C, Zhao Y, Dai G, et al. A novel STFSA-CNN-GRU hybrid model for short-term traffic speed prediction. IEEE Transactions on Intelligent Transportation Systems, 2022, 24(4): 3728–3737 doi: 10.1109/TITS.2021.3117835

[21] Xu C, Ji J, Liu P. The station-free sharing bike demand forecasting with a deep learning approach and large-scale datasets. Transportation research part C: emerging technologies, 2018, 95: 47–60. doi: 10.1016/j.trc.2018.07.013

[22] Fang W, Chen Y, Xue Q. Survey on research of RNN-based spatio-temporal sequence prediction algorithms. Journal on Big Data, 2021, 3(3): 97. doi: 10.32604/jbd.2021.016993

[23] Dudukcu H V, Taskiran M, Taskiran Z G C, et al. Temporal Convolutional Networks with RNN approach for chaotic time series prediction. Applied Soft Computing, 2023, 133: 109945. doi: 10.1016/j.asoc.2022.109945

[24] Li X, Xu Y, Chen Q, et al. Short-term forecast of bicycle usage in bike sharing systems: a spatial-temporal memory network. IEEE Transactions on Intelligent Transportation Systems, 2021, 23(8): 10923–10934. doi: 10.1109/TITS.2021.3097240

[25] Chen Y, Wang W, Hua X, et al. Discrete wavelet transform application for bike sharing system check-in/out demand prediction. Transportation Letters, 2023: 1–12. doi: 10.1080/19427867.2023.2219045

[26] Bai L, Yao L, Wang X, et al. Deep spatial–temporal sequence modeling for multi-step passenger demand prediction. Future Generation Computer Systems, 2021, 121: 25–34. doi: 10.1016/j.future.2021.03.003

[27] Chai J, Song J, Fan H, et al. ST-Bikes: Predicting Travel-Behaviors of Sharing-Bikes Exploiting Urban Big Data. IEEE Transactions on Intelligent Transportation Systems, 2022.

[28] Zi W, Xiong W, Chen H, et al. TAGCN: Station-level demand prediction for bike-sharing system via a temporal attention graph convolution network. Information Sciences, 2021, 561: 274–285. doi: 10.1016/j.ins.2021.01.065

[29] Lee S H, Ku H C. A dual attention-based recurrent neural network for short-term bike sharing usage demand prediction. IEEE Transactions on Intelligent Transportation Systems, 2022, 24(4): 4621–4630. doi: 10.1109/TITS.2022.3208087

[30] Lin L, He Z, Peeta S. Predicting station-level hourly demand in a large-scale bike-sharing network: A graph convolutional neural network approach. Transportation Research Part C: Emerging Technologies, 2018, 97: 258–276. doi: 10.1016/j.trc.2018.10.011

[31] Kim T S, Lee W K, Sohn S Y. Graph convolutional network approach applied to predict hourly bike-sharing demands considering spatial, temporal, and global effects. PloS one, 2019, 14(9): e0220782. doi: 10.1371/journal.pone.0220782 31525227 PMC6746382

[32] Huang Z, Zhang W, Wang D, et al. A GAN framework-based dynamic multi-graph convolutional network for origin–destination-based ride-hailing demand prediction. Information Sciences, 2022, 601: 129–146. doi: 10.1016/j.ins.2022.04.024

[33] Reggiani G, Salomons A M, Sterk M, et al. Bicycle network needs, solutions, and data collection systems: A theoretical framework and case studies. Case studies on transport policy, 2022, 10(2): 927–939. doi: 10.1016/j.cstp.2022.03.006

[34] Liu X, Pelechrinis K. Excess demand prediction for bike sharing systems. Plos one, 2021, 16(6): e0252894. doi: 10.1371/journal.pone.0252894 34138884 PMC8211247

[35] Guo Y, Zhou J, Wu Y, et al. Identifying the factors affecting bike-sharing usage and degree of satisfaction in Ningbo, China. PloS one, 2017, 12(9): e0185100. doi: 10.1371/journal.pone.0185100 28934321 PMC5608320

[36] Yan S, Liu M, O’Connor N E. Parking behaviour analysis of shared e-bike users based on a real-world dataset-a case study in dublin, ireland. 2022 IEEE 95th Vehicular Technology Conference:(VTC2022-Spring). IEEE, 2022: 1-6.

[37] Li X, Xu Y, Zhang X, et al. “Improving short-term bike sharing demand forecast through an irregular convolutional neural network”. Transportation research part C: emerging technologies, 2023, 147: 103984. doi: 10.1016/j.trc.2022.103984

[38] Yang D, Li S, Peng Z, et al. “MF-CNN: traffic flow prediction using convolutional neural network and multi-features fusion”. IEICE TRANSACTIONS on Information and Systems, 2019, 102(8): 1526–1536. doi: 10.1587/transinf.2018EDP7330

[39] Cheadle C, Vawter M P, Freed W J, et al. “Analysis of microarray data using Z score transformation”. The Journal of molecular diagnostics, 2003, 5(2): 73–81. doi: 10.1016/S1525-1578(10)60455-2 12707371 PMC1907322

[40] Song C, Lin Y, Guo S, et al. “Spatial-temporal synchronous graph convolutional networks: A new framework for spatial-temporal network data forecasting”. Proceedings of the AAAI conference on artificial intelligence. 2020, 34(01): 914-921.

[41] Jiang W, Luo J. “Graph neural network for traffic forecasting: A survey”. Expert Systems with Applications, 2022, 207: 117921. doi: 10.1016/j.eswa.2022.117921

[42] Jia Z, Lin Y, Wang J, et al. “Multi-view spatial-temporal graph convolutional networks with domain generalization for sleep stage classification”. IEEE Transactions on Neural Systems and Rehabilitation Engineering, 2021, 29: 1977–1986. doi: 10.1109/TNSRE.2021.3110665 34487495 PMC8556658

